# Using Diffraction Deep Neural Networks for Indirect Phase Recovery Based on Zernike Polynomials

**DOI:** 10.3390/s24020698

**Published:** 2024-01-22

**Authors:** Fang Yuan, Yang Sun, Yuting Han, Hairong Chu, Tianxiang Ma, Honghai Shen

**Affiliations:** 1 Changchun Institute of Optics, Fine Mechanics and Physics, Chinese Academy of Sciences, Changchun 130033, China; yuanfang19@mails.ucas.ac.cn (F.Y.);; 2 University of Chinese Academy of Sciences, Beijing 100049, China

**Keywords:** phase recovery, diffractive deep neural network, Zernike polynomial

## Abstract

The phase recovery module is dedicated to acquiring phase distribution information within imaging systems, enabling the monitoring and adjustment of a system’s performance. Traditional phase inversion techniques exhibit limitations, such as the speed of the sensor and complexity of the system. Therefore, we propose an indirect phase retrieval approach based on a diffraction neural network. By utilizing non-source diffraction through multiple layers of diffraction units, this approach reconstructs coefficients based on Zernike polynomials from incident beams with distorted phases, thereby indirectly synthesizing interference phases. Through network training and simulation testing, we validate the effectiveness of this approach, showcasing the trained network’s capacity for single-order phase recognition and multi-order composite phase inversion. We conduct an analysis of the network’s generalization and evaluate the impact of the network depth on the restoration accuracy. The test results reveal an average root mean square error of 0.086λ for phase inversion. This research provides new insights and methodologies for the development of the phase recovery component in adaptive optics systems.

## 1. Introduction

Distortion phase recovery, as a measurement technique based on optical principles, is utilized for quantitatively describing the phase and amplitude distribution of optical waves during their propagation. It can identify and correct distortions within the system through comparisons with the actual wavefront, thereby enhancing imaging quality and performance. Distortion phase recovery can be applied in a wide range of fields, such as atmospheric optics, laser device manufacturing, and optical communication. Moreover, it plays a crucial role in supporting the development of adaptive optical systems.

Because determining the system’s aberration function properly and quickly is essential to the function of adaptive optics systems, numerous techniques have been devised to accomplish this. In more traditional methods, the aberration function can be directly measured using wavefront sensors like the Shack–Hartmann wavefront sensor [1,2], the curvature wavefront sensor [3], and the shearing interferometer [4] multi-order diffractive optical element [5,6,7]. These wavefront sensors that rely on complex specialized optical hardware devices have the advantages of high precision and good stability. However, they also suffer from drawbacks such as high hardware costs, high computational complexity, and limited scalability. Since the 1990s, artificial neural networks (ANNs) and deep learning have been applied to determine the Zernike coefficients representing a given wavefront [8]. This is because they are capable of learning complex relationships without the need for specific physical rule programming [9,10]. As research has progressed, methods have been developed to directly reconstruct wavefront phases from intensity images using deep learning [11]. Utilizing ANNs, whether directly or indirectly for phase recovery, allows the desired features to be inferred directly from the data, enabling a higher degree of adaptability. However, it is important to acknowledge that traditional neural networks for phase reconstruction come with significant computational costs and memory usage, and their processing speed is slightly inferior to hardware wavefront sensors.

Optical Neural Networks (ONNs) constructed using optical matrices have emerged as a promising alternative for next-generation neural computing [12]. By leveraging the speed of light and massive parallelism of optical signals within a medium, optical networks offer potential solutions to the challenges faced by electronic counterparts, such as computational power and energy consumption. In 2018, Lin et al. introduced an all-optical deep learning framework composed of multiple layers of diffractive surfaces that formed the structure of a deep learning network, referred to as the Diffractive Deep Neural Network (DDNN) [13]. DDNNs can be created via element-wise multiplication by utilizing the interaction between light and matter, where ‘pixels’ on the diffractive surface are similar to ‘neurons’ on network layers; these neurons are then coupled via optical diffraction principles. Diffractive Deep Neural Networks (DDNNs) possess tremendous flexibility [14,15], scalability [16], and significant advantages in terms of their processing power and performance. Subsequently, researchers thoroughly investigated and verified the generalization abilities of DDNNs, using them in a variety of fields, such as scattering imaging [17], gesture classification [18], and orbital angular momentum spectrum measurements [19].

DDNNs have demonstrated promising achievements in the retrieval and correction of optical interference phases. In 2022, Zhao et al. proposed an adaptive optical compensation scheme based on a DDNN to restore the distortions induced by oceanic turbulence on vortex beams [20]. A comparative analysis with wavefront recovery schemes based on CNN and GS algorithms was conducted, revealing that the DDNN achieved the highest improvement in mode purity for compensated vortex beams. Subsequently, the team introduced a Hybrid Optoelectronic Deep Neural Network (HOEDNN), where the DDNN is trained to establish mapping between the distorted orbital angular momentum (OAM) intensity patterns, and the intensity distribution without oceanic turbulence interference [21]. A CNN is then employed to recognize the output of the DDNN. In addition, in 2023, Elena Goi et al. introduced a compact multi-layer diffraction neural network module imprinted on an imaging sensor [22]. This module first focuses the input light through a lens, then reconstructs the Zernike-based pupil phase distribution from the point spread function. By integrating CMOS sensors with diffraction elements, they achieved direct pupil phase recovery based on the superposition of the first 14 orders of Zernike polynomials. Furthermore, in the field of real-time wavefront correction systems, Cui et al. trained the DDNN as a wavefront corrector, validated its correction effect in scenarios such as off-axis and binary stars, and positioned it between the imaging lens and the image plane to improve the wavefront correction frequency [23].

**Figure 1:**
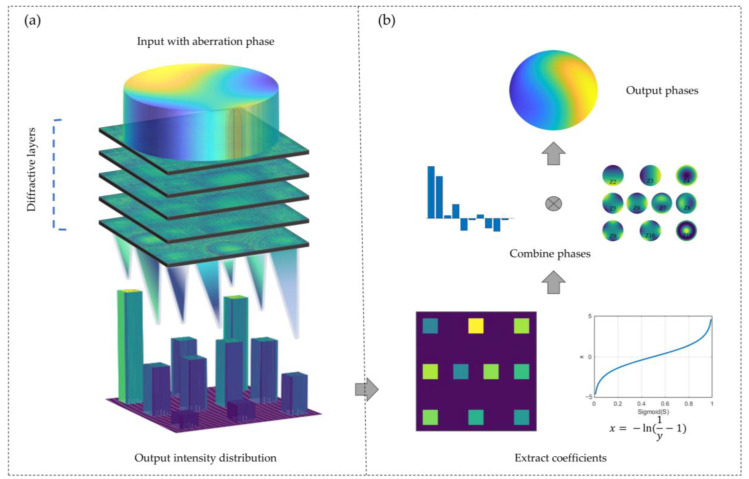
Schematic diagram of the DDNN-based indirect phase recovery scheme. (**a**) Illustration of the beam modulation process in the optical system. When parallel light with an unknown distorted wavefront passes through the pre-trained diffraction layers, a concentrated intensity distribution is obtained in a specific region of the output plane. (**b**) Flowchart for post-processing in the computer. After simple operations such as summing the output plane intensity collected by the imaging module, applying the sigmoid inverse transformation, and combining them, the predicted target phase is obtained.

Existing research predominantly emphasizes end-to-end direct processing methods, yet in the application of optical systems with relatively simple and easily modellable aberration patterns, Zernike polynomials, representing common distortion modes in optical systems, can more effectively and concisely describe wavefront aberrations. To bridge the gap concerning indirect phase retrieval methods within the domain of diffraction neural networks combined with wavefront adaptive optics, we propose an indirect phase retrieval scheme based on Zernike polynomials, as shown in Figure 1. We employ deep learning to train a set of transmissive diffractive layers and achieve the all-optical inversion of the mapping relationship between the distorted phase carried by the input beam and its corresponding Zernike coefficients. After the jointly modulated light, which carries an unknown distorted wavefront, passes through a prepared multilayer diffractive element, the desired intensity distribution is obtained in a specific region of the output plane, as shown in Figure 1a. The intensity within this region corresponds to the coefficients of Zernike polynomials, and a straightforward combination operation yields the distribution of the unknown distorted phase, as shown in Figure 1b. The simulation results indicate an average root mean square (RMS) error of 0.086λ for the phase results obtained via this method, thus meeting the imaging quality requirements of the system. This approach holds great potential for widespread application across various fields in the future.

## 2. Theory and Analysis

### 2.1. Indirect Phase Recovery Scheme Based on Diffraction Neural Network

**Figure 2:**
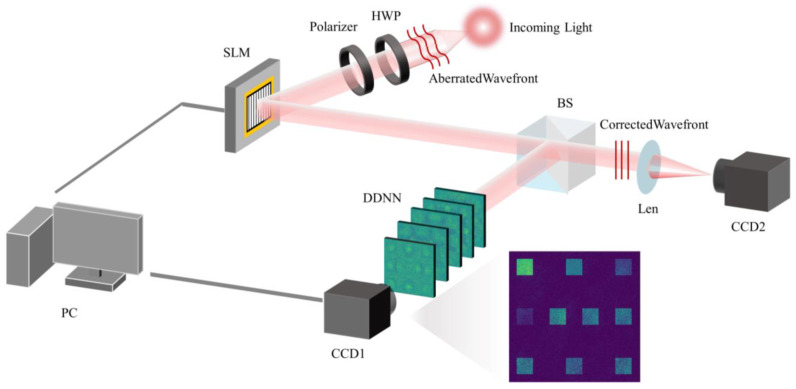
Conceptual diagram of the proposed indirect phase recovery scheme.

The conceptual illustration of our proposed diffraction neural network-based indirect phase recovery scheme is presented in Figure 2. In this system, the wavefront detection module comprises a diffraction network and a Charge-Coupled Device (CCD1), while the wavefront control module is managed by a Personal Computer (PC). The wavefront correction module consists of a polarizer and half wave plate (HWP), used to adjust the polarization direction of the incident beam, and a spatial light modulator (SLM), used for phase modulation. In this scheme, incident beams experiencing distortion due to factors such as fluid (e.g., atmospheric turbulence) or biological tissues have their phases modulated layer by layer through a well-trained multilayer diffraction neural network. The intensity representation of the mapped distorted phase Zernike coefficients is obtained in the output field. Subsequently, the intensity distribution captured by the CCD1, the resolution of which needs to be greater than 400 × 400, is fed into the computer for the reconstruction of the phase screen and reverse operations, resulting in a compensatory phase screen. Finally, the spatial light modulator corrects the beam, effectively compensating for the distorted wavefront. In this scheme, a 50:50 beam splitter (BS) is used to divide the light into two beams. One beam is used to complete the above wavefront detection work, and the other beam is used as the verification beam. The comparison of the point spread function (PSF) before and after correction can be seen through the imaging of the beam on the CCD2 through the lens, which is used to describe the response of the focusing optical imaging system to the point source or point object.

The diffractive neural network is an all-optical machine learning platform that calculates a given task through successive transmission layers, with each diffraction layer typically composed of tens of thousands of diffraction units in order to modulate the phase or amplitude of the incident light. Similar to deep learning techniques, it can learn a certain mapping relationship from a large dataset. Subsequently, through error backpropagation and optimization methods such as stochastic gradient descent, it refines the modulation values of each layer to map the complex-valued input field containing optical information that is of interest to the desired output field. Assuming that the amplitude of a light beam is $A\left(x,\;y\right)$ and its wavefront aberration is $\Phi \left(x,\;y\right)$, where  x and y are polar coordinates on the input pupil plane, the pupil function $P\left(x,\;y\right)$ of the input beam can be expressed as follows:

(1) $$P\left(x,y\right)=A\left(x,y\right)e^{jk\Phi \left(x,y\right)}$$

where $\;k=\frac{2\pi }{\lambda }$ is the wave number and the imaginary unit $j=\sqrt{-1}$.

As has been established, Φ can be expressed as a linear combination of a series of Zernike polynomials, and it is represented as follows:

(2) $$\Phi \left(x,y\right)=\underset{i}{\sum }a_{i}Z_{i}$$

In this expression, $Z_{i}$ represents the i-th Zernike polynomial, and $\;\;a_{i}$ represents the i-th Zernike coefficient.

**Figure 3:**
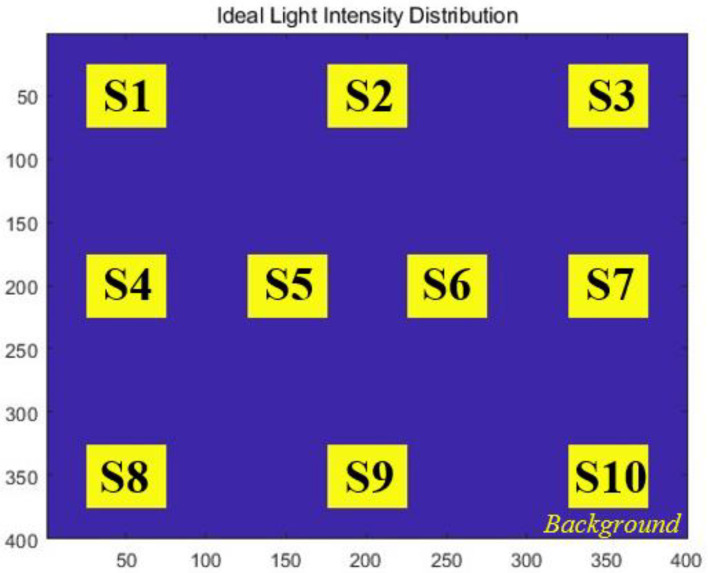
Schematic diagram of output plane assignment method. $S_{i}$ represents the ten areas that are assigned values.

Moreover, since the output plane intensity does not have negative values, in order to allow the positive and negative values of Zernike coefficients to be represented in the output plane as intensities, the coefficients $a_{i}$ undergo a Sigmoid transformation to ensure their distribution within the (0, 1) range, which is expressed as follows:

(3) $$S_{i}\left(x_{i},y_{i}\right)=\frac{1}{1+e^{-a_{i}}}$$

where $S_{i}\left(x_{i},y_{i}\right)$ represents the transformed value of the i-th coefficient; its relationship with the intensity of the output plane is illustrated in Figure 3. $\left(x_{i},y_{i}\right)$ denotes the coordinates of the i-th region in the plane. According to Equations (1) and (2), the complex amplitude of the distorted input beam and the Zernike coefficients a, representing the wavefront aberration, satisfy a certain mapping relationship:

(4) f:P→a

This paper utilizes the learning capability of a diffraction neural network similar to a deep neural network to accurately estimate the mapping f. Equation (3) reveals the relationship between the output field intensity and the coefficients a, which addresses the challenge of expressing the output plane in the negative domain using this transformation.

### 2.2. Network Structure and Parameter

DDNN is a system composed of a series of transmission or reflection diffraction layers and is endowed with the capacity to learn and modulate the optical field. Each layer consists of N × N pixels, where each pixel serves as a sensor node corresponding to neurons in a neural network. Similarly, the complex-valued transmission coefficients (including amplitude and phase) of each pixel in the diffraction element are trainable network parameters. The propagation of light between diffraction layers in the DDNN follows a connectivity pattern that is highly analogous to traditional fully connected neural networks, where each unit in a diffraction layer is connected to all units in the next layer. Each diffraction neuron can be regarded as the starting point of a secondary wave, and the complex-valued transmission coefficients and input field of each neuron jointly determine the amplitude and phase of the secondary wave. The free space propagation (FSP) between adjacent layer neurons adheres to the Rayleigh–Sommerfeld diffraction formula [24]. Therefore, the optical field of the secondary wave can be expressed as follows:

(5) $$w_{i}^{l}\left(x,y,z\right)=\frac{z-z_{i}}{r^{2}}\left(\frac{1}{2\pi r}+\frac{1}{\dot{j}\lambda }\right)\exp\left(\frac{j2\pi r}{\lambda }\right)$$

where $w_{i}^{l}$ represents the i-th neuron unit located at the position $\left(x_{i},y_{i},z_{i}\right)$ in the l-th layer of the DDNN. $r=\sqrt{{\left(x-x_{i}\right)}^{2}+{\left(y-y_{i}\right)}^{2}+{\left(z-z_{i}\right)}^{2}}$ is the distance from the starting point to this neuron. λ is the wavelength.

The transmission coefficients $t_{i}^{l}\left(x_{i},y_{i},z_{i}\right)$ of layer l can be represented by the amplitude and phase terms, formulated as follows:

(6) $$t_{i}^{l}\left(x_{i},y_{i},z_{i}\right)=a_{i}^{l}\left(x_{i},y_{i},z_{i}\right)\exp\left(j\varphi_{i}^{l}\left(x_{i},y_{i},z_{i}\right)\right)$$

This paper considers the ideal scenario of a pure phase-type DDNN structure, where $a_{i}^{l}\left(x_{i},y_{i},z_{i}\right)=1$; this neglects the optical loss. According to the Huygens–Fresnel principle, the incident wave at layer l is the coherent superposition of the secondary waves emitted by each unit in layer l−1 [25]. Therefore, the complex amplitude $n_{i}^{l}$ of the light field output by the i-th neuron at position $\left(x_{i},y_{i},z_{i}\right)$ in layer l can be expressed as follows:

(7) $$n_{i}^{l}\left(x_{i},y_{i},z_{i}\right)=w_{i}^{l}\left(x,y,z\right)\cdot t_{i}^{l}\left(x_{i},y_{i},z_{i}\right)\cdot \Sigma_{k}n_{k}^{l-1}\left(x_{i},y_{i},z_{i}\right)$$

In this equation, $\Sigma_{k}n_{k}^{l-1}\left(x_{i},y_{i},z\right)$ is the sum of the outputs of all sensor nodes in layer l−1, which, after propagating to layer l, undergoes phase modulation via the i-th neuron in layer l. The secondary wave from the previous layer diffracts to the next layer, eventually diffracting to the output layer.

The fully connected structure of the network requires a high level of connectivity between the diffraction elements in each layer. The derived model of the second-order wavefront field is valid only when there is sufficient transmission of information between layers, i.e., when the diffracted light outputs of each layer can be fully interconnected. Therefore, before designing the network parameters, it is necessary to calculate the diffraction angles of the light passing through the diffractive neurons, ensuring that the next layer of diffractive optical elements can be fully covered. The maximum half-cone diffraction angle can be calculated using the Fraunhofer diffraction formula. When the diffraction order is minimized, the expression for the maximum half-cone angle is as follows:

(8) $$\theta_{max}=\sin^{-1}\left(\frac{\lambda }{2d_{f}}\right)$$

where $d_{f}$ represents the size of the diffractive neurons in this model.

It is evident that a combination of larger wavelengths and smaller neurons yields a larger half-cone diffraction angle. Therefore, in previous studies, terahertz lasers were commonly used as light sources. In this paper, a He-Ne laser with a wavelength of 632.8 nm was employed as the light source, following the general approach used to design the visible light diffraction neural networks mentioned in reference [26]. For a square diffraction layer, it is necessary to ensure that the radius r of each diffraction point is greater than the side length w of each diffraction layer. This ensures that the entire region of the next layer’s diffractive elements is covered by the output light field of the previous layer. The side length w of the diffraction layer can be expressed in terms of the number of neurons N and the size $d_{f}\;$ of the diffractive neuron. The diffraction radius is determined by both the maximum half-cone diffraction angle and the interlayer distance d. The physical quantity relationships included in the above model can be summarized as Equation (9).

(9) $$\left\{\begin{array}{l} r\geq w\\ w=\sqrt{N\cdot {d_{f}}^{2}}\\ r=d\cdot \tan\theta_{max} \end{array}\right.$$

By combining this with Equation (8), it can be deduced that the interlayer distance d  must satisfy the following inequality:

(10) $$d\geq \sqrt{N}\cdot d_{f}\cdot \sqrt{\frac{4{d_{f}}^{2}}{\lambda^{2}}-1}$$

**Figure 4:**
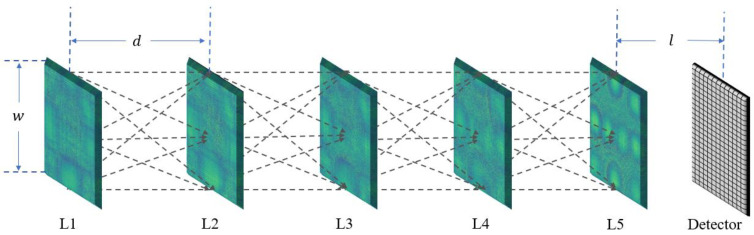
Schematic diagram of the network structure.

As shown in Figure 4, the diffraction neural network proposed in this paper for use in indirect phase recovery consists of 5 layers of diffractive elements. Each layer has 400 × 400 pixels, and each pixel has a size of 4 μm. Without considering pixel gaps, the size of the square diffraction layer is 3.2 mm. After calculation, the distance between adjacent diffraction layers, *d*, is set to 20 mm. Considering the requirement for the intensity of the received plane, the distance l between the last diffraction layer and the CCD is set as 10 mm.

### 2.3. Network Backpropagation

In order to ensure consistency between the network output and the target output, the Adam gradient descent optimizer is employed during the training process to adjust the network weights and minimize the loss function. The loss function in this paper consists of two parts. Firstly, it is assumed that the input data for training are $X\;\in \;\left\{X^{1},\;X^{2},\;\ldots ,\;Xn\right\}$, where X represents the distorted wavefront phase. The true values for each input data correspond to an array $Ai=\left\{ai^{1},\;ai^{2},\;ai^{3},\;ai^{4},\;ai^{5},\;ai^{6},\;ai^{7},\;ai^{8},\;ai^{9},\;ai^{10}\right\}$, where the elements represent the coefficients of the first ten Zernike polynomials. In the training set, the ground truth can be represented as $\;A\;\in \;\left\{A^{1},\;A^{2},\;\ldots ,\;An\right\}$. The mapping relationship between the distorted phase and the true coefficients is denoted as $F_{model}\left(X\right)\to \;A$. Performing the prediction task for these data using the diffractive optical neural network system involves modulating the distorted wavefront phase in the input data onto a coherent light beam, obtaining the input field $U_{in}$, and then collecting the intensity result $I_{out}$ of the output field via a photoelectric coupling device.

The training dataset comprises the mapping relationship $F_{model}(X)\to A$, where X represents the input data with a distorted wavefront phase. In the optical system, the desired mapping relationship is $\;F_{model}(U_{in})\to I_{out}$. These two mapping relationships share a common essence at their core but can be considered as two different approaches during approximation, guiding the network convergence and progressively optimizing the phase parameters $\varphi_{layer}$ of diffractive neurons. Consequently, two distinct loss functions are derived: the RMS error between the output plane intensity $I_{out}$ and the ideal output intensity ${\hat{I}}_{out}$, and the RMS between the computed actual coefficient array $\hat{A}$ and the target coefficient array A in the training set. Mathematically, these can be expressed as follows:

(11) $$Loss_{1\;}=\underset{\varphi_{layer}}{\min}\sqrt{\frac{1}{n}\underset{n}{\sum }{\left|{\hat{I}}_{out}-I_{out}\right|}^{2}},\varphi_{layer}\in \left\{0,2\pi \right\}\;Loss_{2}=\underset{\varphi_{layer}}{\min}\sqrt{\frac{1}{n}\underset{n}{\sum }{\left|\hat{A}-A\right|}^{2}},\varphi_{layer}\in \left\{0,2\pi \right\}$$

*Loss*_1_ represents the loss of the diffractive neural network’s output plane light intensity, which serves to avoid the presence of stray light spots in the background of the output field, thus achieving overall optimization. *Loss*_2_ is the loss of label accuracy in the neural network’s phase inversion effect, which plays a role in controlling the intensity of the effective region on the output plane. Therefore, the combined total loss of the network can be expressed as follows:

(12) $$Loss=Loss_{1\;}\times Loss_{2}$$

## 3. Datasets and Network Training

To assess the performance of the diffraction neural network-based indirect phase recovery approach, we employed the Zernike polynomial method to simulate phase aberrations, thus generating a substantial volume of wavefront data for the training and validation datasets. To independently validate the network model’s effectiveness in addressing tasks related to single Zernike aberrations and combined Zernike aberrations, two distinct datasets were created for training, each consisting of single Zernike aberrations and combined Zernike aberrations.

Dataset 1: Phase distortions generated from single Zernike polynomials ranging from Z1 to Z10 were utilized in this study. The ground truth consisted of Zernike coefficients, which were individually transformed using Equation (3) to obtain values ranging between zero and one. These transformed coefficients represented the intensity values assigned to the corresponding output regions. The transformation was applied only to non-zero coefficients to avoid interference from zero terms. Dataset 1 comprised 10,000 training images and 2000 testing images. The training set consisted of 10 different individual Zernike polynomials, each with 1000 images, while the testing set included 200 images for each polynomial.

Dataset 2: The distorted phase is generated by combining Zernike polynomials of orders one to ten. The output ground truth is obtained by transforming the coefficients of each Zernike term through Equation (3), resulting in output intensity values within the range zero–one. Dataset 2 comprises a total of 12,000 images, with 10,000 images in the training set and 2000 images in the testing set.

**Figure 5:**
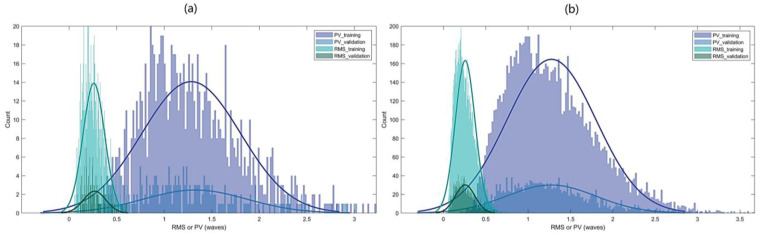
Statistic graph of wavefront errors of two datasets. (**a**) By calculating the distribution mean of 10 categories of single Zernike polynomials in Dataset 1, the RMS and PV distributions of 1000 training data and 200 test data are obtained. (**b**) RMS and PV distributions of 10,000 training data and 2000 test data in Dataset 2.

For most applications of adaptive optics (AO) systems, the wavefront errors typically fall within a specific range over a given time frame. Drawing from accumulated experience in astronomical observations, we set the peak-to-valley (PV) values of the input aberration phases within the range of 0.3λ to 3λ, with an average PV value of 1.5λ and an average RMS error of 0.25λ. The dataset comprises a total of 12,000 images generated using Zernike polynomials, from orders two to eleven. Among these, 10,000 images constitute the training set, and the remaining 2000 images form the test set. The distribution of the aforementioned data is illustrated in Figure 5.

**Figure 6:**
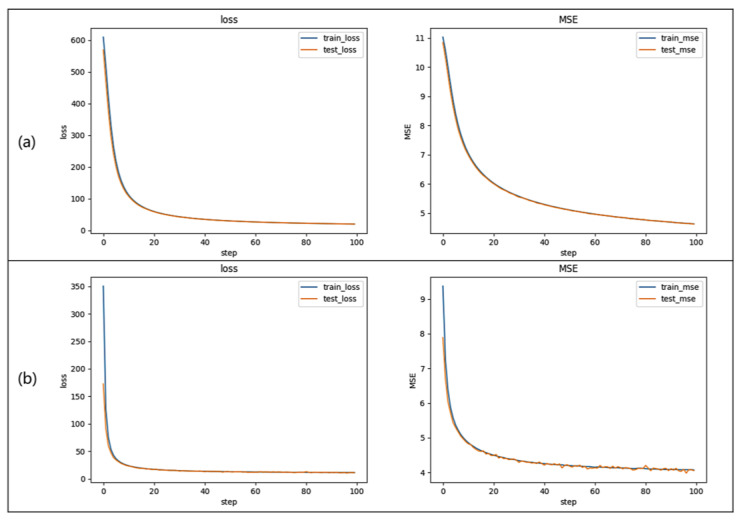
The loss function and MSE decline curve. (**a**) Network training process based on combined Zernike wavefront distortion data. (**b**) Network training process based on single Zernike wavefront distortion data.

The training of the diffraction neural network model for indirect phase recovery was conducted using the PyTorch 3.6 framework on an NVIDIA GeForce RTX 3080 GPU with 12 GB of RAM. The training environments and parameters for the two networks were consistent. The Adam optimizer was employed to optimize the parameters of the diffraction neural network, with training conducted over 100 epochs using a batch size of 128 and a learning rate of 0.01. The loss function and mean square error (MSE) decline curve of the training set and verification set during the training process are shown in Figure 6. The total duration for a single training session was 8 h.

**Figure 7:**
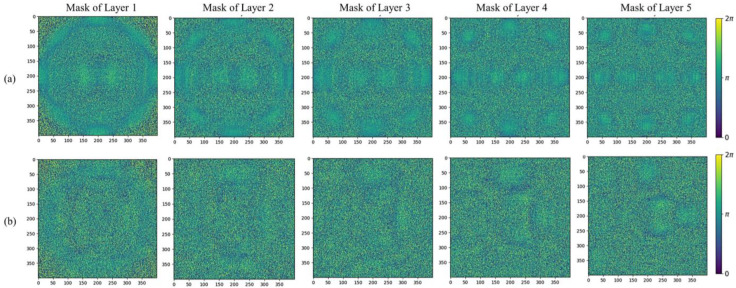
The predicted phase distributions for each diffraction layer obtained after training. (**a**) Combined Zernike wavefront distortion training results. (**b**) Single Zernike wavefront distortion training results.

We constructed a five-layer diffraction neural network to learn the mapping relationship between the distorted phase and the decomposition coefficients of the Zernike polynomials. The input beam is represented in the form of complex amplitude, while the output is expressed as the distribution of light intensity. The training process involves the continuous adjustment of the phase values for each pixel in the five diffraction layers and aims to progressively minimize the differences between the predicted 10 Zernike coefficients and the ground truth. The phase distribution results for each layer after training are depicted in Figure 7. Subsequently, the physical preparation of the diffraction layers can be accomplished using techniques such as photolithography or 3D printing based on the obtained phase distributions for each layer.

## 4. Performance Evaluation

### 4.1. Testing for Non-Degenerate Response in the Network

First, the network is trained with the data of the Z7 part of the single coma in Dataset 1, and it is tested whether the network is non-degenerate when single-order aberration is input. The objective of this was to assess the system’s ability to generate reliable and stable outputs when confronted with different input scenarios. In particular, in situations in which the input phase has the same amplitude but opposite signs, it is crucial to ensure that the network can produce the corresponding coefficient values.

**Figure 8:**
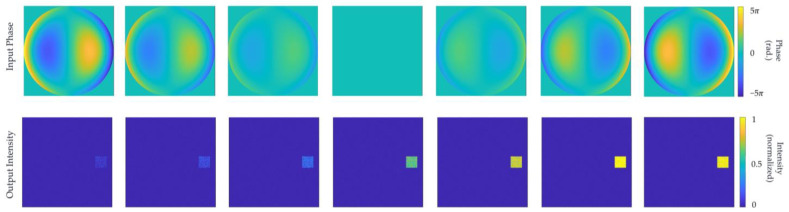
Output plane intensity when scanning the magnitude of a single-order aberration input phase within the range of $\left[-3\lambda ,\;3\lambda \right]$.

Seven sets of vertical astigmatism term pupil phases, with the seventh Zernike phase (Z7) having magnitudes scanned within the range of $\left[-3\lambda ,\;3\lambda \right]$, are imposed on a collimated beam with unit intensity. The acquisition of the seven phases involves using the positive aberrations within the range of [−λ, λ] as the basis, and applying 0, ±1, ±2, and ±3 multiplication transformations to each of them, respectively. The resulting intensity distribution in the output plane after modulation that was performed by the diffraction network is illustrated in Figure 8. The test results indicate that altering the sign and amplitude of the input phase yields spots of different sizes at the location corresponding to the seventh term in the output plane. This demonstrates the network’s sensitivity to variations in the input and simultaneously validates its robustness, ensuring its reliability in practical applications.

**Figure 9:**
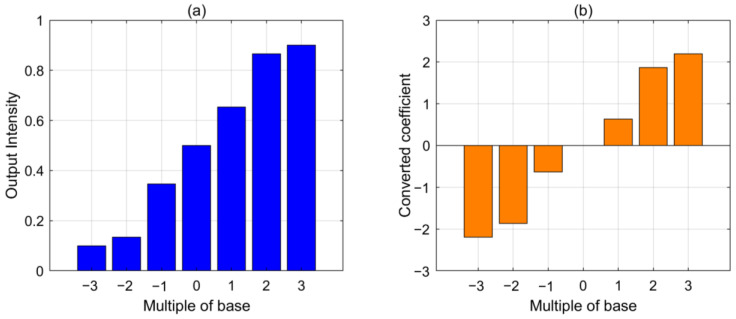
Analysis of non-degenerate response test results. (**a**) Correspondence between output intensity and multiple of base phase. (**b**) Correspondence between the transformed predicted coefficient and multiple of base phase.

To illustrate the relationship between input phase magnitude and output light intensity, a bar graph was constructed, plotting the mean intensity extracted from the targeted region of the network’s output plane against the base phase multiplier as the horizontal axis, as shown in Figure 9a. As the multiplier increases, the obtained light intensity also increases, demonstrating that the proposed approach can produce different output values in response to input wavefront phases of varying magnitudes. Subsequently, an inverse transformation, given by Equation (3), was applied to the obtained intensity values to restore the corresponding Zernike coefficients for phase generation. Similarly, a bar graph was plotted with the base phase multiplier as the horizontal axis, revealing that the distribution of the restored coefficients corresponds to the input wavefront’s peak-to-valley (PV) value and keeps consistent with the positive and negative directions. As the phase range tested exceeds that of the network training set, this discussion focuses on the corresponding trends between phase and coefficients, without considering specific numerical relationships.

### 4.2. Testing the Single-Order Zernike Identification Function

**Figure 10:**
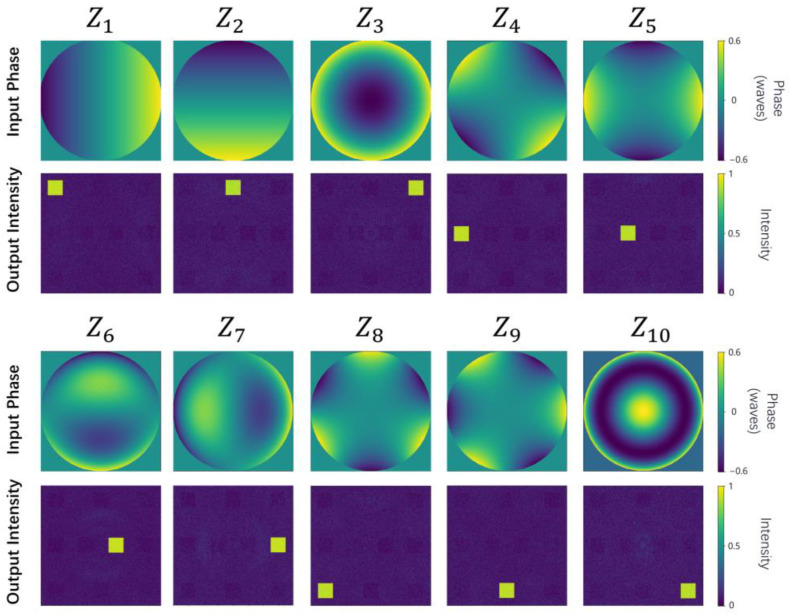
Results of single-order Zernike aberration recognition test for the first ten orders.

Benefiting from a partitioned, multi-category network output design, the system demonstrates the capacity for classification recognition when subjected to single-order Zernike aberrations as the input. For the network trained with Dataset 1, distorted phase distributions corresponding to the first ten orders of Zernike aberrations, with phase magnitudes ranging from −0.6λ to 0.6λ, are individually superimposed onto the input beam. After modulation and propagation through the network, concentrated spots appear in the regions corresponding to the respective terms in the output plane, with the intensity in other regions approaching zero. As depicted in Figure 10, the test results unequivocally confirm the network’s ability to accurately identify the categories of the first ten orders of Zernike aberrations. This capability enhances the ease of diagnosing issues in optical systems with specific aberrations, thus aiding in the implementation of corresponding measures in order to improve and optimize the system.

### 4.3. Combined Phase Retrieval Performance Test

**Figure 11:**
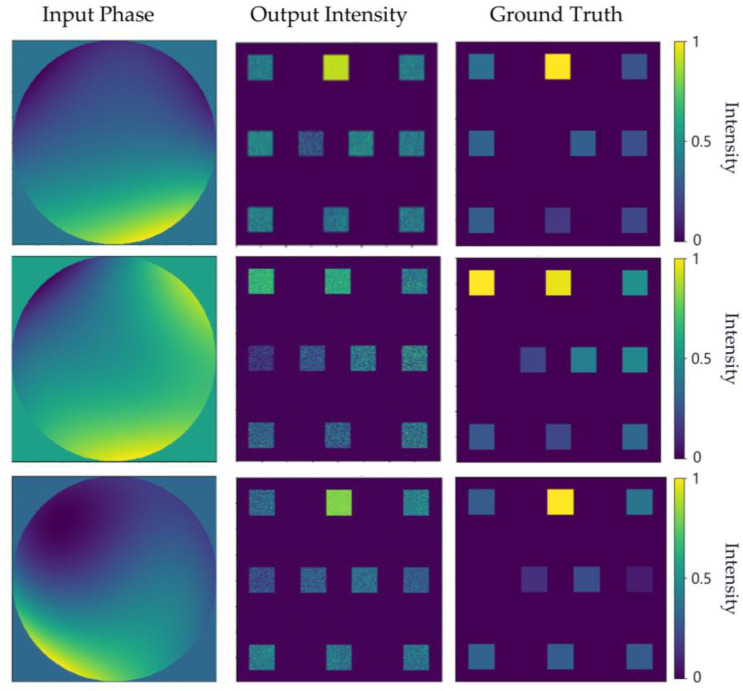
The combined phase of the input, the intensity of the output plane, and the corresponding true value of the output plane.

**Figure 12:**
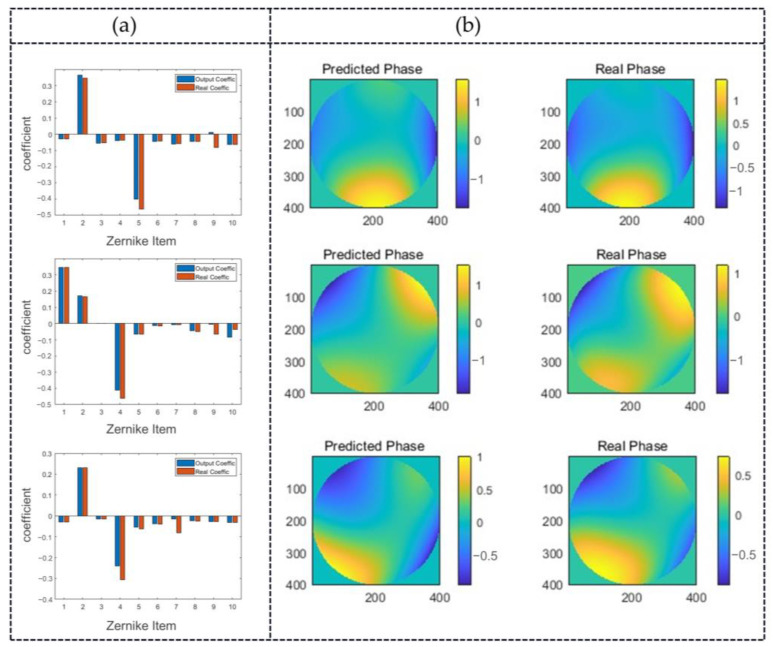
Analysis of network output results. (**a**) Zernike polynomial coefficients obtained after transformation of the output intensity and comparison with true coefficients. (**b**) Comparative analysis of predicted results and ground truth.

In order to further assess the performance of the diffraction neural network-based indirect wavefront phase retrieval scheme, the output intensity distribution was obtained using the first ten combined Zernike phases as input, as shown in Figure 11. A subjective evaluation of the results reveals that spots corresponding to larger coefficients are brighter, while those corresponding to smaller coefficients are darker, demonstrating a correspondence between the output and the true values. From an objective perspective, the coefficient distribution obtained via averaging and the inverse operations on the intensity within specific regions is illustrated in Figure 12a.

**Figure 13:**
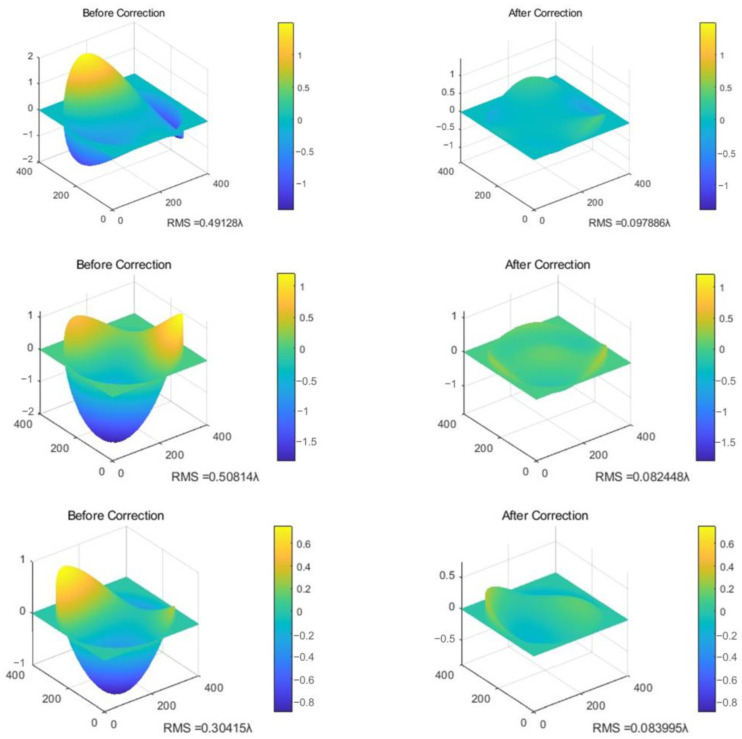
Comparison of original phase and corrected residual phases.

Utilizing the network output, the reconstruction of the wavefront is performed by obtaining the first ten Zernike coefficients ($a_{out}$). These coefficients are then substituted into Equation (2). The reconstructed phase distribution, denoted as $\Phi_{out}$, is shown in Figure 12b. To further validate the accuracy of the reconstructed phase, the conjugate of $\Phi_{out}$ is superimposed with the input phase. This process simulates the ideal aberration correction using mathematical calculations, and the corrected result is depicted in Figure 13. The graph illustrates the change in the RMS error before and after correction. In addition, Figure 13 also illustrates the comparison between the original phase and the residual wavefront after correction is performed by the predicted phase on the same coordinate scale.

A comparison reveals a significant reduction in the RMS after correction, indicating that the system is capable of recovering the combined aberration phases of the first ten Zernike orders. Moreover, the error in the recovered phase falls within an acceptable range. Thus, after testing the network’s capacity for inversion using 1000 test data, the average RMS error of the output results is determined to be 0.086λ. According to the Maréchal criterion for assessing the optical system quality, the corrected system imaging quality is considered.

### 4.4. Influence of the Number of Layers on the Network

**Figure 14:**
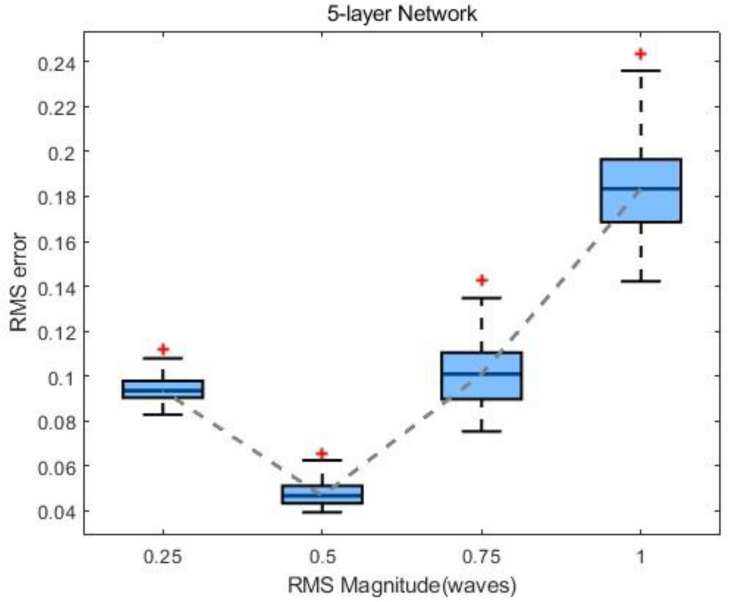
Generalization test results of the five-layer network. The red + represents outliers, and the gray dotted line represents the line connecting the medians of each group of data.

During the network training process, the dataset employed includes phase values distributed within a predefined range. For data beyond this phase distribution range, the network’s accuracy during phase recovery cannot be precisely guaranteed. Therefore, it is crucial to validate the network’s generalization performance. A test set, scanning the RMS within one wavelength, is used to evaluate the network; this comprises four test groups, each containing 200 randomly generated phases within a specified range. The RMS error values of the test results are calculated, and the distribution box plot is illustrated in Figure 14. It is observed that the network exhibits minimal restoration error for the input-phase RMS sizes at 0.5λ. The distortion wavefront restoration error for the input-phase RMS sizes in the range of 0–0.75λ can be controlled around 0.1λ. In conclusion, the effective application range of this scheme is within 0.75λ, demonstrating the good generalization performance of the network within this range.

**Figure 15:**
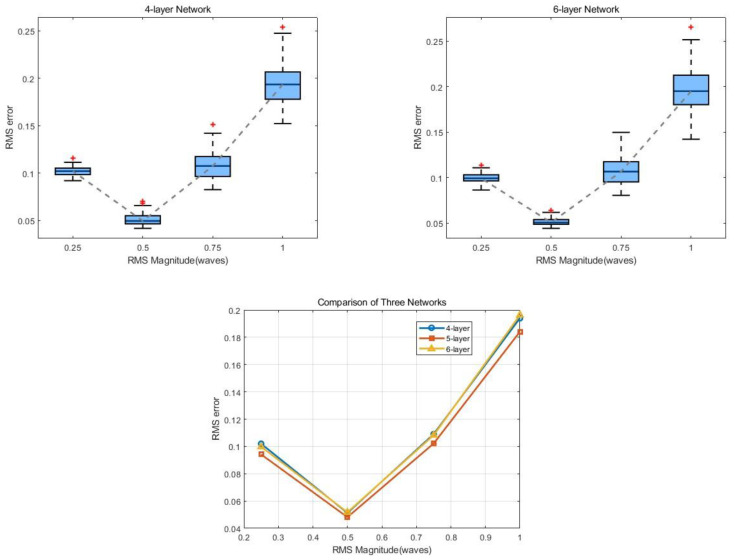
Generalization test results of 4-layer and 6-layer networks, with comparison of test results for networks with different numbers of layers. The red + represents outliers, and the gray dotted line represents the line connecting the medians of each group of data.

In addition, it has been established that increasing the number of layers in traditional neural networks typically confers various benefits, such as an enhanced expressive power, an improved capacity for generalization, and a reduced risk of overfitting. Therefore, we aim to examine the impact of varying the number of layers on the accuracy of the network output results. Initially, the number of diffraction layers is set to four and six while the other parameters are kept constant, and the network is retrained accordingly. The resulting four-layer and six-layer network models are then tested for the occurrence of RMS error in the output results using the same method; the test results for networks with different numbers of layers are illustrated in Figure 15.

The comparison of RMS errors during the process of phase reconstruction performed by networks with different numbers of layers reveals that, when the input phase size is 0.5λ, the three networks exhibit a consistent capacity for inversion. However, as the input phase size deviates from the training set range, the five-layer network demonstrates the best phase reconstruction accuracy among the three, indicating its superior capacity for generalization. Consequently, the five-layer network is chosen as the final design parameter for this approach.

## 5. Conclusions

To address the deficiency in the indirect phase recovery of diffraction neural networks and to specifically target the distortion phase recovery problem in optically simple and easily modellable systems with aberration modes, we propose a diffraction neural network-based indirect phase inversion scheme in this work. This scheme utilizes the passive diffraction of multiple layers of diffraction units to achieve the inversion of distorted phases corresponding to the Zernike polynomial coefficients within a specific range. The mathematical model and mapping relationships of the DDNN are derived, and the DDNN model is trained to obtain the optimal solution for the diffraction layer phase distribution that meets the phase modulation requirements. When a distorted beam is incident on the diffraction network, the trained model will output the Zernike coefficients corresponding to the distorted phase. The simulation results demonstrate that this scheme can achieve single-order Zernike phase identification and recover combined phases within a specific range. The evaluation of the network is conducted via the output coefficients and phase correction results, proving that the network significantly reduces the mean square error of the distorted phase, thus greatly improving the imaging quality of optical systems. Additionally, the simulation verifies the impact of the number of diffraction layers on the network performance. By endowing the diffraction neural network with wavefront sensing capabilities, this work achieves low-power, high-speed phase recovery, exhibiting advantages with regard to convenience and cost control over the Shack–Hartmann sensor in traditional adaptive optics systems. The proposed wavefront recovery scheme, based on diffractive neural networks, demonstrates superior performance in terms of power consumption and real-time processing compared to wavefront recovery schemes relying on deep learning networks. However, the complexity of the recovered wavefront in this scheme is constrained by the designed light intensity distribution on the output plane, limiting its effectiveness in addressing the recovery of high-order complex phases. Further research is required to explore aspects such as the physical implementation and performance enhancement of this scheme. In summary, this scheme provides a new approach for distorted wavefront recovery, and future optical experiments are expected to validate and contribute to the development of adaptive optics systems.

## Data Availability

Data are contained within the article.

## References

[1] Dubey N., Kumar R., Rosen J. (2021). COACH-based Shack–Hartmann wavefront sensor with an array of phase coded masks. Opt. Express.

[2] Hu L., Hu S., Gong W., Si K. (2019). Learning-based Shack-Hartmann wavefront sensor for high-order aberration detection. Opt. Express.

[3] Roddier F. (1988). Curvature sensing and compensation: A new concept in adaptive optics. Appl. Opt..

[4] Wyant J.C. (1975). Use of an ac heterodyne lateral shear interferometer with real–time wavefront correction systems. Appl. Opt..

[5] Porfirev A.P., Khonina S.N. (2016). Experimental investigation of multi-order diffractive optical elements matched with two types of Zernike functions. Proc. SPIE.

[6] Khonina S.N., Karpeev S.V., Porfirev A.P. (2020). Wavefront aberration sensor based on a multichannel diffractive optical element. Sensors.

[7] Khorin P.A., Porfirev A.P., Khonina S.N. (2022). Adaptive Detection of Wave Aberrations Based on the Multichannel Filter. Photonics.

[8] McGuire P.C., Sandler D.G., Lloyd-Hart M., Rhoadarmer T.A. (1999). Adaptive optics: Neural network wavefront sensing, reconstruction, and prediction. Scientific Applications of Neural Nets: Proceedings of the 194th WE Heraeus Seminar Held at Bad Honnef, Germany, 11–13 May 1998.

[9] Ma H., Liu H., Qiao Y., Li X., Zhang W. (2019). Numerical study of adaptive optics compensation based on convolutional neural networks. Opt. Commun..

[10] Möckl L., Petrov P.N., Moerner W.E. (2019). Accurate phase retrieval of complex 3d point spread functions with deep residual neural networks. Appl. Phys. Lett..

[11] Cumming B.P., Gu M. (2020). Direct determination of aberration functions in microscopy by an artificial neural network. Opt. Express.

[12] Peurifoy J., Shen Y., Yang Y., Jing L., Cano-Renteria F., Joannopoulos J., Tegmark M., Soljačić M. (2017). Nanophotonic Inverse Design Using Artificial Neural Network. Front. Opt..

[13] Lin X., Rivenson Y., Yardimci N.T., Veli M., Luo Y., Jarrahi M., Ozcan A. (2018). All-optical machine learning using diffractive deep neural networks. Science.

[14] Luo Y., Mengu D., Yardimci N.T., Rivenson Y., Veli M., Jarrahi M., Ozcan A. (2019). Design of task-specific optical systems using broadband diffractive neural networks. Light Sci. Appl..

[15] Kulce O., Mengu D., Rivenson Y., Ozcan A. (2021). All-optical information-processing capacity of diffractive surfaces. Light Sci. Appl..

[16] Liu C., Ma Q., Luo Z., Hong Q., Zhang H., Miao L., Yu W., Cheng Q., Li L., Cui T. (2022). A programmable diffractive deep neural network based on a digital-coding metasurface array. Nat. Electron..

[17] Li Y., Luo Y., Mengu D., Bai B., Ozcan A. (2023). Quantitative phase imaging (QPI) through random diffusers using a diffractive optical network. Light Adv. Manuf..

[18] Zhou Y., Shui S., Cai Y., Chen C., Chen Y., Abdi-Ghaleh R. (2023). An improved all-optical diffractive deep neural network with less parameters for gesture recognition. J. Vis. Comun. Image Represent..

[19] Wang H., Zhan Z., Hu F., Meng Y., Liu Z., Fu X., Liu Q. (2023). Intelligent optoelectronic processor for orbital angular momentum spectrum measurement. PhotoniX.

[20] Zhan H., Peng Y., Chen B., Wang L., Wang W., Zhao S. (2022). Diffractive deep neural network based adaptive optics scheme for vortex beam in oceanic turbulence. Opt. Express.

[21] Zhan H., Wang L., Wang W., Zhao S. (2023). Hybrid opto-electronic deep neural network based orbital angular momentum mode recognition scheme in oceanic turbulence. JOSA B.

[22] Goi E., Schoenhardt S., Gu M. (2022). Direct retrieval of Zernike-based pupil functions using integrated diffractive deep neural networks. Nat. Commun..

[23] Pan X., Zuo H., Bai H., Wu Z., Cui X. (2023). Real-time wavefront correction using diffractive optical networks. Opt. Express.

[24] Yevick D., Thylén L. (1982). Analysis of gratings by the beam-propagation method. J. Opt. Soc. Am..

[25] Goodman J.W. (1995). Introduction to Fourier Optics.

[26] Chen H., Feng J., Jiang M., Wang Y., Lin J., Tan J., Jin P. (2021). Diffractive deep neural networks at visible wavelengths. Engineering.

